# A Dental Gathering—1893

**Published:** 1890-07

**Authors:** 


### EDITORIAL.

### A Dental Gathering—1893.

The question of holding a meeting of dentists in 1893, at the same time and place of the World's Columbian Exposition is being somewhat discussed. Exactly what kind of a meeting should be held upon that occasion, or what should be done, is yet to be decided.

It would seem eminently proper, however, that the initiative for such a gathering should be taken at an early date in order that the best plans might be devised, arrangements made and carried out. It should be planned and provided for upon a scale in harmony with the spirit of the occasion, and such as would produce the most complete and satisfactory results.

Since the enterprise should be initiated in this country it would seem that the two largest representative bodies of dentists in the country might start the ball rolling. In just what way this initiative step should be taken, of course, is matter for consideration by these bodies should the subject come before them. If there is a pure interest and earnestness in this matter there will be no difficulty.

It has been suggested that this might be a memorial meeting of the American Dental Association. A question might arise as to the propriety of giving it this trend since it would be desirable to bring into co-operation an entire harmony with the great assembly of other societies of this country and colleges as well, and there are men in the profession in this, and perhaps in other countries, who, for some reason or other, have not thus far identified themselves with any association of dentists, and yet who would desire to co-operate in a meeting of this kind, and moreover, whose presence it would be desirable to have. It should also be made so cosmopolitan in character that dentists of all nations would regard it as desirable to be present.

This great meeting should not be handicapped with any organization which has gone before it, or that may come after it. It should be truly and clearly *sui generis*. It should be made as nearly as possible to harmonize with the spirit of the time and the great occasion on which this is to be held.

We would suggest, therefore, that it is eminently proper that the American Dental Association and the Southern Dental Association, the two chief representative dental bodies in this country, should take the initiative in this matter. And we trust that in the meantime this subject may have the thoughtful consideration of all well-wishers of dental science and art.

---

At the meeting of the Michigan State Dental Society the principal points in Dr. Crouse's remarks were, first, that by combining in some such an organization as the Dental Protective Association we banded the strength of ten thousand men into one, and all defence necessary can be made with but little more expense than would be required for an individual. The preparation of our case will answer for all, and all evidence can be collected better by an association than in any other way. Second, that it is very important that all get into the association at this time for the reason that an appeal suit to the Supreme Court, before the association was formed, will probably be decided in favor of the Crown Company, owing to deficiency of evidence and imperfect presentation. If the Crown Company gain this suit they will send notices all over the United States, thereby demoralizing the profession and causing many to pay them money who should be in the Protective Association and avoid that calamity.

The Protective Association will be ready with new evidence and a new record and can take care of the members against any claims of the Crown Company. If you are not in the association the association will not take care of you when the time comes.

If each man in the profession will pay ten dollars and assume a responsibility of ten more, without any further assessments, we will have an organization that is sure to break up all this abuse and save the dental profession an annual outlay of, on an average, $100 each to unjust claimants. Remember it will cost more than ten dollars to join later, and will certainly cost more than that if you do not protect yourself against unjust claims.

